# Does severe hypoxia during irradiation of *Aedes aegypti* pupae improve sterile male performance?

**DOI:** 10.1186/s13071-022-05577-0

**Published:** 2022-11-28

**Authors:** Dylan A. Tussey, Kenneth J. Linthicum, Daniel A. Hahn

**Affiliations:** 1 Department of Entomology & Nematology, University of Florida, 1881 Natural Area Drive, Gainesville, FL 32608 USA; 2 Center for Medical, Agricultural, and Veterinary Entomology, United States Department of Agriculture, 1600 SW 23rd Drive, Gainesville, FL 32608 USA; 3 Department of Entomology, University of California Riverside, 9240 S. Riverbend Ave, Parlier, CA 93658 USA

**Keywords:** Sterile insect technique, Hypoxia, *Aedes*, Mating competitiveness

## Abstract

**Background:**

The yellow fever mosquito, *Aedes aegypti*, vectors several pathogens responsible for human diseases. As a result, this mosquito species is a priority for control by mosquito control districts in Florida. With insecticide resistance development becoming a concern, alternative control strategies are needed for *Ae. aegypti*. Sterile insect technique (SIT) is an increasingly popular option that is being explored as a practical area-wide control method. However, questions about sterile male performance persist. The objectives of this study were to determine the extent to which hypoxia exposure prior to and during irradiation effects the longevity, activity and mating competitiveness of sterile male *Ae. aegypti*.

**Methods:**

Male longevity was monitored and analyzed using Cox regression. Mosquito activity was recorded by an infrared beam sensor rig that detected movement. Competing models were created to analyze movement data. Fecundity and fertility were measured in females mated with individual males by treatment and analyzed using one-way ANOVAs. Mating competition studies were performed to compare both hypoxia and normoxia treated sterile males to fertile males. Competitiveness of groups was compared using Fried’s competitiveness index.

**Results:**

First, we found that subjecting *Ae. aegypti* pupae to 1 h of severe hypoxia (< 1 kPa O_2_) did not directly increase mortality. One hour of hypoxia was found to prevent decreases in longevity of irradiated males compared to males irradiated in normoxic conditions. Exposure to hypoxia prior to irradiation did not significantly improve activity of sterile males except at the highest doses of radiation. Hypoxia did significantly increase the required dose of radiation to achieve > 95% male sterility compared to males irradiated under normoxic conditions. Males sterilized after an hour in hypoxic conditions were significantly more competitive against fertile males compared to males irradiated under normoxic conditions despite requiring a higher dose of radiation to achieve sterility.

**Conclusions:**

Hypoxia was found to greatly improve key performance metrics in sterile male *Ae. aegypti* without any significant drawbacks. Little work other than increasing the target dose for sterility needs to be conducted to incorporate hypoxia into SIT programs. These results suggest that SIT programs should consider including hypoxia in their sterile male production workflow.

**Graphical Abstract:**

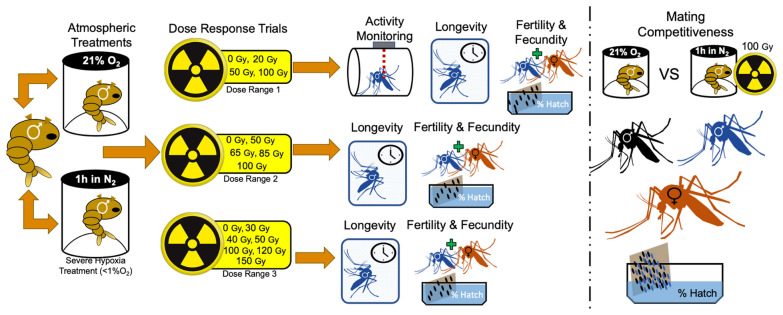

**Supplementary Information:**

The online version contains supplementary material available at 10.1186/s13071-022-05577-0.

## Background

*Aedes aegypti* is an important target for mosquito management because of its capacity to transmit numerous pathogens that can cause human diseases, including yellow fever, dengue, chikungunya and Zika viruses [1–3]. Aside from potentially vectoring these pathogens, *Ae. aegypti* is a major nuisance in urban areas because this mosquito is anthropophilic, living primarily near human dwellings, and is capable of breeding in small containers—from gutters and old tires to plastic bags, bottle caps and other detritus [2, 4, 5]. *Aedes aegypti* presents a challenge for chemical control because they breed in cryptic locations and often reside in and near homes where insecticidal sprays are less likely to reach [6]. In addition, many species of mosquitoes, especially important vector species, are becoming increasingly resistant to commonly used insecticides like organophosphates and pyrethroids [7–9]. Within Florida, many populations of *Ae. aegypti* are showing development of resistance to pyrethroids in highly urbanized areas, such as Miami and Orlando [9–11]. These large cities with international airports are particularly vulnerable to the spread of mosquito-borne diseases [12–14]. As a result of the mounting issues with conventional control of disease-vectoring mosquitoes, there is an urgent need to develop and sustain tactics beyond conventional chemical control for *Ae. aegypti*, including efforts to create viable area-wide control programs, such as sterile insect technique [6, 15].

Sterile insect technique (SIT) is a biological control method that implements the release of sterilized male insects to mate with wild females, resulting in population reduction through infertility [16, 17]. Traditional SIT through the use of ionizing radiation was first developed for the eradication of primary screwworm (*Cochliomyia hominivorax*) in the US in the 1950s [18, 19]. From 1962 through 1998, the United States Department of Agriculture, in agreements with Mexico and Central American countries, eradicated primary screwworm in North and Central America with a buffer zone in Panama, where production facilities still produce sterile males for release [20]. In 2016–2017, primary screwworm was found infesting deer and companion animals in the Florida Keys but was rapidly re-eradicated using SIT [21]. SIT programs are currently used in the US near ports of entry to prevent the establishment of agricultural pests such as Mediterranean fruit fly in Florida and California and the Mexican fruit fly in the Rio Grande Valley of Texas [22–24].

There is active interest in developing SIT programs to control *Aedes* spp. in urban areas worldwide [25–28]. While SIT can be an effective method of area-wide control, a critical facet of successful SIT programs is the production of high-quality males with excellent mating competitiveness against wild males [29, 30]. Mosquitoes, like all other insects, are susceptible to damage and stress at multiple steps in the SIT production and distribution pipeline that can potentially result in sterile males with reduced mating competitiveness [26, 31–33]. Low-quality sterile males with poor mating competitiveness can increase the cost of control or prevent effective control entirely [29, 34]. Therefore, it is important to develop methods that produce high-quality sterile mosquitoes to give budding SIT programs the best chance of success.

One potential way to improve the performance of irradiated male insects is to expose them to a secondary stressor that can induce a hormetic response. Hormesis can be defined as the process by which exposure to a small quantity of a stressor improves subsequent performance, despite the fact that exposure to larger quantities of the same stressor will decrease subsequent performance [35, 36]. Hormetic responses typically involve exposure to a mild dose of one stressor leading to enhanced protection against a larger dose of the same stressor [37, 38]. However, mild exposure to one stressor can also induce greater protection to subsequent exposure to other stressors, a process called cross-tolerance [39–41]. Rapid stress hardening is a type of hormetic conditioning that can occur quickly, where brief exposures to a stressor will lead to enhanced resistance to future, more intense occurrences of that stressor or cross-resistance to other stressors [42, 43].

A commonly used hormetic stressor for insects that are sterilized using radiation is to place insects in low-oxygen environments [44–46]. For example, hypoxic conditioning that initially occurred unintentionally in the production of sterile Mediterranean fruit fly (*Ceratitus capitata*) pupae has been found to increase survival to adulthood and improve flight performance and mating success in SIT programs when hypoxia-treated males are compared to medfly males irradiated in normal, oxygen-rich atmospheres [44, 47, 48]. Thus, irradiation in hypoxia is a critical step in many fruit fly SIT programs worldwide [44, 47, 49].

Because both radiation and hypoxia generate oxidative stress, it is thought that conditioning insects to hypoxia induces biochemical and cellular mechanisms that enhance performance by mitigating radiation stress [45, 50–52]. Consistent with this hypothesis, López-Martínez and Hahn [45] found that brief exposures to severe hypoxic conditions (< 1 kPa O_2_) prior to irradiation increased antioxidant capacity in Caribbean fruit flies (*Anastrepha suspensa*). Higher antioxidant capacity was also correlated with increased flight and mating activity [45]. A subsequent study found that anoxic conditioning prior to and during irradiation significantly increased the lifespan of irradiated male *A. suspensa* and increased the sexual performance of sterile male flies throughout their lifespan [53]. Similarly, tsetse flies (*Glossina pallidipes*) were found to have a significantly longer lifespan when irradiated in anoxic conditions without impacting sterility [54]. Hypoxic conditions prior to or during irradiation have also been found to increase survival and field performance. For example, severe hypoxia pre-treatments improved the longevity, flight performance and mating success of cactus moths (*Cactoblastis cactorum*) in both the laboratory and a field-release trial [46].

Incorporating hypoxic stress during irradiation to induce a hormetic response could potentially be a simple and cost-effective method for improving the quality of sterile male mosquitoes, yet research on potential effects of hypoxia during irradiation on mosquitoes is sparse, and while hypoxia does appear to confer some resistance to radiation damage, there is no consensus on what lifelong performance benefits hypoxia treatment can confer for sterile males in a SIT program. Irradiation in nitrogen atmospheres was found to offer protection of somatic tissues in *Culex quinquefasciatus* pupae and adult males, requiring a higher dose of radiation to induce sterility, but showed no improvement in male competitiveness once sterility was achieved [55]. In contrast, Hallinan and Rai [56] found that irradiating male *Ae. aegypti* pupae or adults after 30 min in nitrogen required a higher dose of radiation to achieve full sterility, yet nitrogen treatments improved the mating ability of males compared to males irradiated at normal oxygen levels. Similarly, Yamada et al. [57] found that irradiation in severe hypoxia (< 0.5% O_2_) required higher doses of radiation to induce sterility in male *Ae. aegypti*, *Ae. albopictus* and *Anopheles arabiensis*. If irradiation in severe hypoxia (< 1 kPa O_2_) can improve the performance of sterilized males, it could be beneficial to incorporate severe hypoxia exposure in *Ae. aegypti* SIT programs. The objectives of this study were to first determine an appropriate duration for exposure of *Ae. aegypti* late-stage pupae to severe hypoxic conditions (< 1 kPa O_2_) to induce a hormetic response without directly reducing survival. We then tested the extent to which severe hypoxia treatments before and during pupal irradiation impacted survival after irradiation, longevity, adult activity, mating performance and sterility across a range of radiation doses, extending the previous observations of Hallinan and Rai [56] and Yamada et al. [57].

## Methods

### Insects

All insects used in these experiments were derived from a colony of *Aedes aegypti* originating from eggs collected from multiple sites in St Johns County, FL, USA, in late 2016 by personnel from the Anastasia Mosquito Control District. After the initial collection of eggs, no supplemental infusions of wild mosquitoes were introduced to the colonies. Colonies have been managed by the USDA-ARS-CMAVE facility in Gainesville, FL, since 2017, maintained in overlapping generations. All adults were caged in 30 × 30 × 30-cm nylon mesh cages (Bugdorm-4E3030, MegaView Science Co., Taiwan) and provided a 10% sucrose solution diet and 150 ml of water to consume ad libitum. Females were provided citrated bovine blood for egg development. Larvae were reared in 5.7-l plastic tubs (Sterilite Corp., Townsend MA, USA) at an approximate density of 700 larvae/l, under constant 28 °C and 12:12 LD photoperiod. Larvae were fed fish food (Tetramin Tropical Flakes, Spectrum Brands Pet LLC, Blacksburg, VA, USA) ad libitum, with water being replaced if algal or fungal growth was observed. Pupae were collected and separated using a larval-pupal separator (John W. Hock Co., Model 5412), with a visual secondary inspection by checking the terminal abdominal segment under a stereo-microscope before being used in experiments.

### Determining hypoxia treatments

**Fig. 1:**
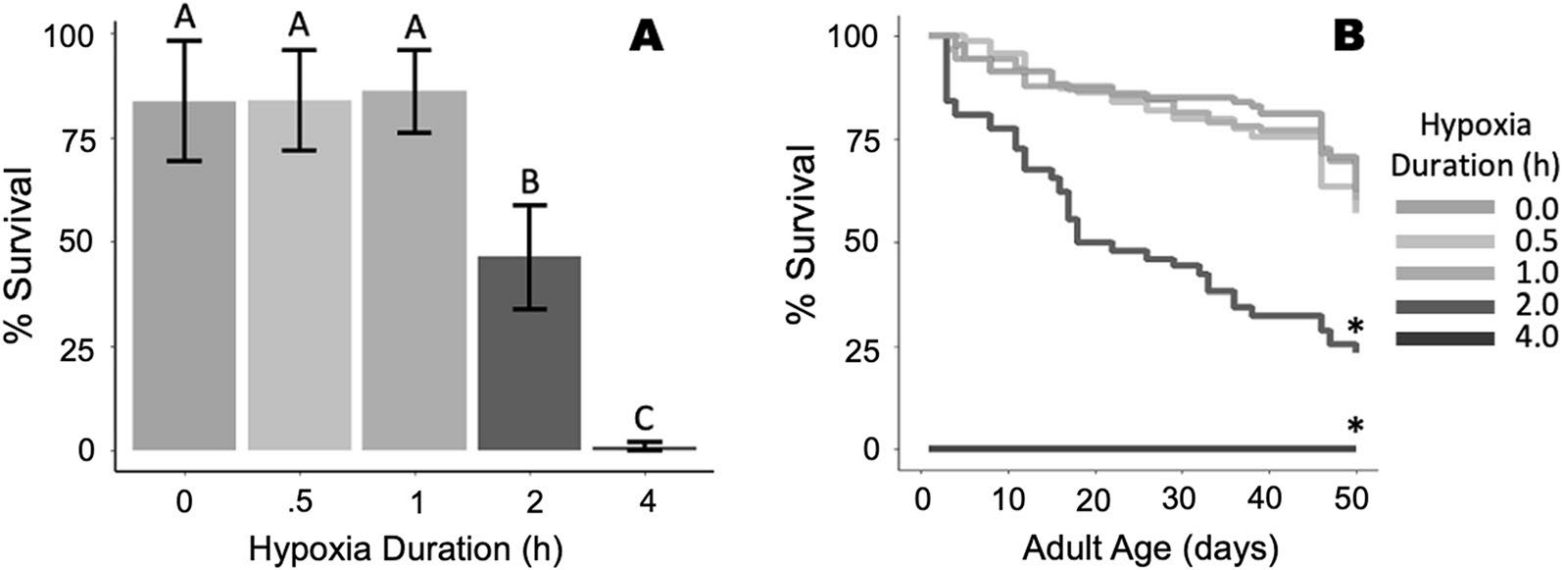
**A** Severe hypoxia did not impact the survival of *Aedes aegypti* late pupae for durations up to 1 h. No late pupae successfully emerged after 4 h of severe hypoxia exposure. **B** Longevity of adult male *Aedes aegypti* was similar among control, 0.5 h and 1 h of severe hypoxia exposure as pupae

A preliminary experiment was conducted to determine an appropriate duration of severe hypoxia to subject pupae in all experiments. Groups of 50 male pupae, 24–36 h old, were placed in petri dishes (60 × 15 mm, Fisher Scientific, Hampton, NH, USA) with moist filter paper to prevent desiccation. Petri dishes were then placed individually into sealed ~ 473 ml polypropylene jars (Paper Mart, Orange, CA, USA) fitted with two three-way, leur-lock valves (Cole-Parmer, Vernon Hills, IL, USA) on the lid to purge the jars (Fig. 1). The containers were flushed with nitrogen (industrial grade, Airgas Co., Radnor PA) for 2 min at a flow rate of approximately 20 l/min, long enough to displace all oxygen in the containers four times. Pupae were then left in these conditions for 0.5, 1, 2 or 4 h. Oxygen sensor patches (RedEye RE-xx-4, Ocean Optics, Largo, FL) were placed inside containers and monitored using a NeoFox-GT fluorometer (Ocean Optics, Largo, FL) to confirm that hypoxic conditions in the containers were sustained for the duration of treatments. We found that all but one container remained in hypoxic conditions throughout experiments. The one container that did not maintain hypoxic conditions was excluded from the study after exposure. An additional 50 pupae were placed in a container without a lid as a normoxic control.

At the termination of the severe hypoxia treatments, pupae were removed from the containers, and petri dishes were filled with 20 ml water to allow them to finish adult development and emerge. Pupae were assessed for survival and adult emergence 3 days after hypoxia treatments by counting dead pupae, pupal exuviae and dead adults that did not fully emerge from pupal exuviae. Adults were held in 30 × 30 × 30-cm screen cages and provided 10% sucrose solution and 150 ml water to feed and drink ad libitum. Adult longevity was recorded by counting daily mortality to determine a suitable duration of severe hypoxia that did not significantly impact the survival of the male mosquitoes. This experiment was replicated four times. At the end of this preliminary experiment, 1 h of severe hypoxia was determined to be an appropriate duration to induce a response in late pupae without impacting mortality, and 1 h was used as our standard time of exposure to hypoxia throughout the rest of the study (Fig. 1).

### Hypoxia and radiation treatments

Male mosquito pupae, 24–36 h old, were placed in 60 × 15-mm petri dishes with moist filter paper to prevent desiccation. Pupae that underwent an hour of severe hypoxia were exposed in sealed ~ 473 ml polypropylene jars as described above. Pupae that were kept in normoxic conditions were irradiated in similar containers without lids. Severe hypoxia treatments were performed by flushing the containers with nitrogen for 2 min, enough time to replace the volume of the container more than four times. Irradiation was conducted using a Cs-137 gamma irradiator (Gammator M, Radiation Machinery Corporation) at a dose rate of ~ 8.8 Gy/m. The timing of severe hypoxia was set to end at the termination of radiation treatment. For example, 50-Gy treatments needed 5 min 41 s in the irradiator to reach the target dose, so pupae were placed in the irradiator after 54 min in nitrogen-flushed conditions to keep all severe hypoxia exposures at 1 h. At the end of irradiation, containers were opened almost immediately to restore normoxic conditions to pupae. Gafchromic HD-V2 film dosimeters (1 cm × 1 cm) (Ashland, Covington, KY, USA, uncertainty < 2%) were individually placed in small paper envelopes and affixed to the bottom of petri dishes. Alanine pellet dosimeters (lot: T030901, Far West Technology, CA, USA, uncertainty 3.5%) were also used with the film dosimeters side by side periodically to confirm the absorbed dose. Alanine pellets were read later at the National Center for Electron Beam Research Texas A&M, College Station, TX. Film was read with a DoseReader 4 spectrophotometer (Radiation General Instrument Development and Production Ltd., Budapest, Hungary) at a wavelength of 590 nm amber light ~ 24 h after irradiation. Absorbed doses of radiation were calculated from the film dosimeters on the bottom of each petri dish irradiated at each target dose.

### Radiation dose-response trials

To test for effects of hypoxia and radiation on male *Ae. aegypti*, three ranges of radiation doses were selected, and several downstream male performance metrics were assessed, including longevity, activity and mating performance. As our research progressed our initial results suggested that we needed a broader range of radiation doses than we had initially estimated to ensure adequate male sterility when pupae were irradiated in hypoxia. Thus, we performed three different trials, each of which had a slightly different range of doses with the maximum dose used increasing from our first trial to our third and last trial. The first dose range trial consisted of 24–36-h-old pupae exposed to target doses of 0, 20, 50 or 100 Gy of radiation in either a normoxic or severe hypoxia atmospheric treatment (< 1 kPa O_2_). In this set of experiments, longevity, daily activity, sterility and mating ability were tested across all eight treatments. The second dose range trial was similar to the first but consisted of pupae exposed to target doses of 0, 50, 65, 85 or 100 Gy radiation doses in either a normoxic or severe hypoxia atmospheric treatment. Males in this dose range were subjected to the same experiments as the first set, with the exception that daily activity monitoring was not performed. A third radiation dose range trial was applied to determine the dose at which severe hypoxia-exposed males would exceed 95% sterility and directly compare their performance to males sterilized by irradiation in normoxia. Because early results found that males irradiated in severe hypoxia retained fertility at higher radiation doses compared to males irradiated in normoxic conditions, we applied different dose ranges to the normoxic vs. severe-hypoxic treatments in this third trial. Male pupae radiation doses in this third dose range trial were as follows: normoxia-treated males received 0, 30, 40 or 50 Gy and severe hypoxia-treated males received 0, 50, 100, 120 or 150 Gy. In this third dose range trial, longevity, sterility and mating competency assays were replicated four times.

### Male longevity

Male longevity assays were identical in all three dose range trials throughout this study. To test for effects of hypoxia and radiation on male *Ae. aegypti* performance, petri dishes containing 50 male pupae were exposed to the combinations of atmospheric and radiation treatments described above. After radiation treatments, dishes were filled with 20 ml water and placed in 30 × 30 × 30-cm mesh cages and pupae were allowed to emerge for 3 days before pupal mortality was assessed. Pupae that had not emerged or had dead adults still partially in pupal exuviae were considered dead. All empty pupal exuviae were counted to confirm emerged adults, and remaining pupae were considered dead. Once adults had emerged, three males from each treatment were removed for mating trials and four males were removed for activity trials, while the remaining ~ 40 males were monitored for longevity.

Adults in longevity treatments were placed in 30 × 30 × 30-cm cages as described above and provided a petri dish with 25 ml water and a 59-ml plastic cup (Solo p200n, Dart Container Corp., Mason, MI) with cotton soaked in a 10% sucrose solution. Water dishes were filled three times weekly, while sucrose solution was filled once weekly, with cotton replaced when it appeared discolored. Mortality was assessed by counting and collecting dead adults from cages daily for at least 5 days each week. Cages were maintained until < 10% of the initial population of adults remained alive. Longevity experiments were replicated five times for dose range 1 and three times for dose range 2 and 3.

### Male activity

Only mosquitoes in the first dose range trial were assessed for activity. Males used for the daily activity study were collected from treatment cages 3 days after emergence. Four males from each treatment were placed individually in 15 × 2.2-cm glass containment tubes sealed on each end with cotton. A 15-ml conical centrifuge tube filled with a 10% sucrose solution was placed on one end of each glass containment tube. A total of 32 glass containment tubes were placed on an activity monitor (Trikinetics LAM25, Waltham, MA, USA) for 11 days. Recorded data used for analysis began 6 h after mosquitoes were placed in the monitor, so they would have time to acclimate to the glass containment tubes and monitor, and subsequently run for an additional 10 days after the starting time. This experiment was repeated three times for a total of 12 replicates for each treatment.

### Mating performance

**Fig. 2:**
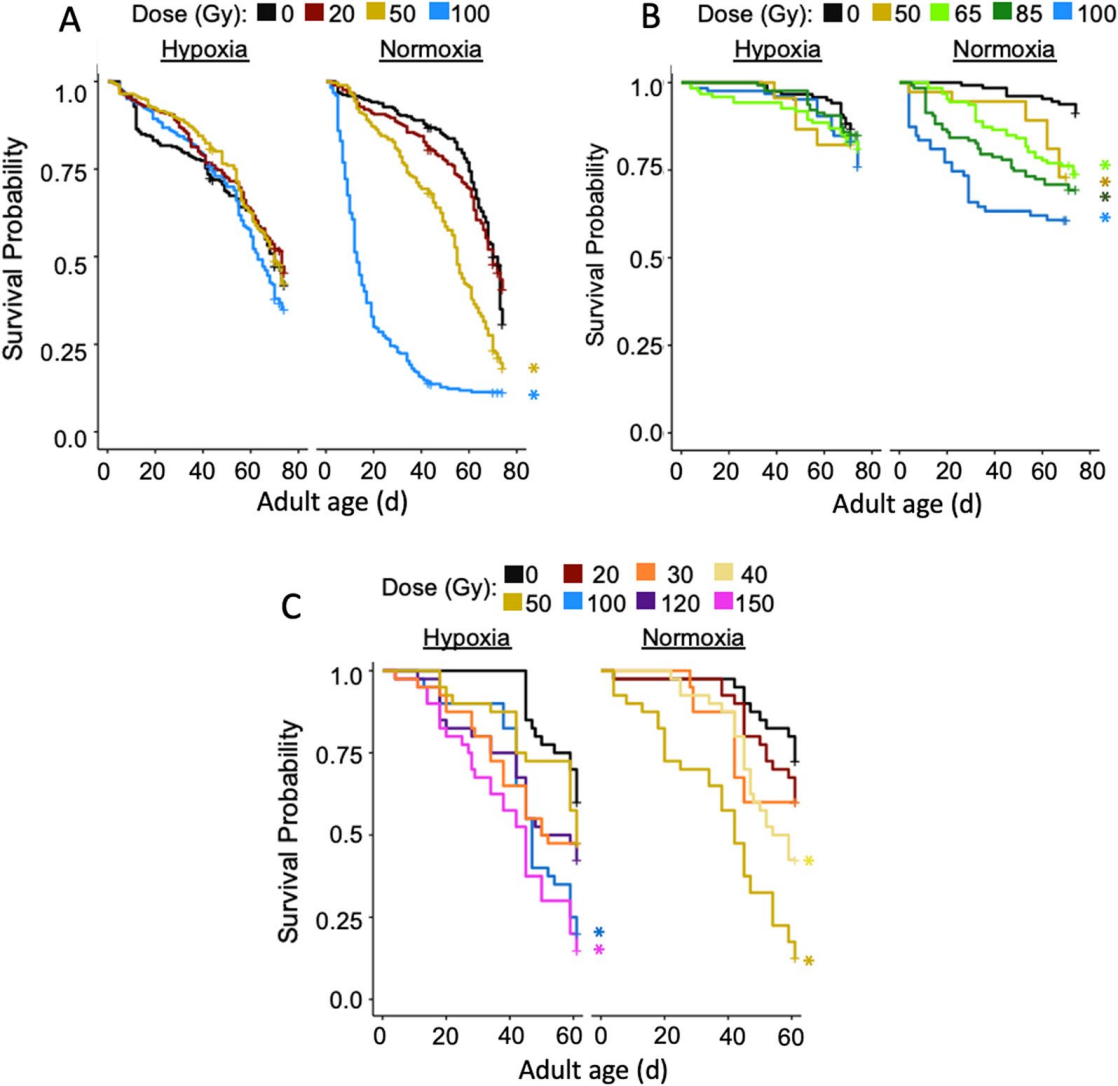
Kaplan-Meier survival curves for males irradiated under different atmospheric conditions for radiation dose ranges 1 (**A**), 2 (**B**) and 3 (**C**). Asterisks indicate significant differences from the control group

Male mating ability and sterility were tested using the following methods for the first and second dose range trials only. Females used in mating trials were collected as pupae from colony stock at the same time as males collected for experiments and allowed to develop and emerge in the absence of males to ensure virginity. Groups of 120 female pupae were placed in a 100 × 25-mm petri dish (Fisher Scientific, Hampton, NH, USA) filled with 100 ml water within a 30 × 30 × 30-cm mesh cage and allowed to emerge. Adult females were provided with water and cotton soaked in 5% sucrose for 3 days after emergence. The sucrose-soaked cotton was removed from the cage at the end of the third day to encourage females to take blood meal on the 4th day after emergence. Females were provided a lambskin condom with 25 ml warmed (~ 40 °C) beef blood 4 days after emergence and allowed to feed for 1 h. After the blood meal, the sucrose-soaked cotton was returned to the cage. Mating trials began 5 days after adult emergence. On the day of mating trials, females were anesthetized with CO_2_ briefly to separate females into groups of three, and each of these groups of three females was placed into a 50-ml conical centrifuge tube (Thermo Scientific, Rochester, NY, USA) for transfer to mating cages. For each treatment, single males were captured individually in 50-ml conical centrifuge tubes by hand to avoid the use of any anesthesia. Each individual male was caged with three blood-fed virgin females for 92 h in modified 1.9-l plastic containers (Leaktite, Leominster, MA, USA) fitted with an oviposition cup with moist seed germination paper for oviposition. Single-male assays were replicated three times within each treatment across four cohorts in dose range 1 and 2 times within each treatment across five cohorts in dose range 2 (Fig. 2).

Egg sheets were collected after 7 days in mating cages in the first and second dose-response trials, placed individually in petri dishes (60 × 30 mm, Fisher Scientific, Hampton, NH, USA) and allowed to dry for 1 week inside an incubator set to 28 °C. Egg sheets were then photographed for eggs to be counted using ImageJ software. A nutrient broth was made to stimulate egg hatching (0.7 l deionized water, 0.25 g of Tetramin tropical fish food, 0.05 g dry activated yeast) and allowed to sit open in the laboratory on a stirring plate for 24 h to encourage microbial growth. Egg sheets were placed in 50-ml petri dishes (100 × 20 mm) with 30 ml of nutrient broth to hatch. Larvae were counted 48 h after being placed in hatching medium before being transferred to larger 100-ml petri dishes and allowed to develop to the pupal stage, where larval mortality was calculated.

Subsequently, males in the third dose range trial were tested using single-pair crosses in a follow-up experiment to determine the dose at which severe hypoxia-exposed males would become > 95% sterile. In this experiment, individual female pupae and treated male pupae were placed in 50-ml petri dishes with deionized water within 950-ml containers. Pupae were allowed to emerge and were provided 10% sucrose solution as a diet. Seven days after adult emergence, females were provided a blood meal, and a strip of paper towel was added to the petri dish of water to serve as an oviposition sheet. Oviposition sheets were collected from each cage 7 days after blood meals and dried for 1 week before counting eggs and determining hatching using the procedures described above. This experiment was replicated four times with three cages per treatment in each replicate, for a total of 12 cages for each treatment.

### Sterile male competitiveness

To determine the efficacy of severe hypoxia treatments on mating competitiveness of sterilized males against unsterilized wild males, cages were set up with a 1:1:1 ratio of unirradiated males, unirradiated females and irradiated males. Based on previous fertility data, sterilized males were either dosed with 50 Gy of radiation in normoxic conditions or 100 Gy of radiation in severe hypoxia so both groups of treated males would have similar sterility levels. Control cages containing males of each treatment at a 2:1 ratio of males to females were used to confirm the fertility status of unsterilized and sterile males.

Cages were established by placing cups with 50 ml deionized water containing male and female pupae inside. Male pupae that underwent irradiation were immediately placed in cages after radiation exposure. Cages were provided fresh water and a 10% sucrose solution as described above. Blood meals were provided to females 7 days after cages were established. Two oviposition cups made from 50-ml plastic cups lined with paper towels were placed in each cage to collect eggs. Oviposition cups were collected 7 days after blood meals. Egg sheets were dried for 1 week before being photographed to count eggs. Egg sheets were then placed in 50-ml petri dishes filled with nutrient broth to stimulate egg hatching. Larvae were collected and counted 48 h after hatching to determine fertility. Mating competition experiments were replicated seven times.

### Data analysis

R software (version 4.0.2) was used to conduct all data analyses. Longevity data were analyzed using Cox regressions for survival. Trikinetics-based activity data were analyzed by calculating the mean daily maximum activity rate. Competing models were then constructed, and an exponential distribution was used to find the maximum likelihood estimate (MLE) of each activity grouping. The log-likelihood function was calculated for each activity grouping within the models, and the Akiake information criterion (AIC) was calculated for each model. Models were then compared, and the model with the lowest AIC value was selected. Differences among severe hypoxia vs. normoxia and radiation dose combinations in mating trials were tested using two-way ANOVA after checking that the data met the assumptions of ANOVA. Both egg and hatch percentage data were checked for outliers using Dixon’s Q tests. Outliers were omitted from formal analyses. Uncensored data and analysis can be found in supplementary information. Fried’s competitiveness index (C) was used to compare the mating performance of sterilized males against wild-type males [58]. Fried’s competitiveness index is calculated as:

$$C = \left( {{W \mathord{\left/ {\vphantom {W S}} \right. \kern-\nulldelimiterspace} S}} \right) \times \left[ {{{\left( {H_{{\text{w}}} - H_{{\text{c}}} } \right)} \mathord{\left/ {\vphantom {{\left( {H_{{\text{w}}} - H_{{\text{c}}} } \right)} {\left( {H_{{\text{c}}} - H_{{\text{s}}} } \right)}}} \right. \kern-\nulldelimiterspace} {\left( {H_{{\text{c}}} - H_{{\text{s}}} } \right)}}} \right]$$

where *W* = the number of wild-type males, *S* = the number of sterile males, *H*_w_ = percentage of egg hatch from wild-type females mating with only wild-type males (control cages), *H*_c_ = the percentage of egg hatch from wild-type females caged with both sterile and wild-type males, and *H*_s_ = the percentage of egg hatch from wild-type females following mating with sterile males only. Higher *C* values indicate increased competitiveness of sterile males against wild-type males, but this index can also be used to compare the competitiveness of one treatment versus another (e.g. males irradiated in normoxia vs. males irradiated in hypoxia).

## Results

### Duration of hypoxia

Male pupae kept in severe hypoxia for durations up to 1 h suffered no significant reductions in survival to adulthood, while exposure durations of 2 h resulted in nearly 50% mortality (*F*_4,5_ = 11.34, *P* = 0.010) (Fig. 1A). No pupae survived to adulthood after exposure to 4 h in severe hypoxic conditions. Adults that experienced severe hypoxic conditions as pupae had similar longevities to the control when exposure was ≤ 1 h, while 2 h of hypoxia resulted in decreased longevity (*z* = 6.42, *P* < 0.001) (Fig. 1B). Given these results, a 1 h exposure to severe hypoxia was used in all subsequent experiments.

### Male longevity

Longevity of males exposed to severe hypoxia in the first dose range trial did not significantly differ from the control group regardless of radiation dose, while males that were dosed with 50 or 100 Gy of radiation in normoxic conditions lived significantly shorter durations than the control group (*χ*^2^ = 555.4, df = 7, *P* < 0.001) (Fig. 2A). In the second dose range trial, males exposed to doses ≥ 50 Gy in normoxic conditions lived significantly shorter durations than control males: *χ*^2^ = 61.02, df = 9, *P* < 0.001) (Fig. 2B). Males in the third dose range trial performed similarly to previous experiments, with shorter longevities as dose of radiation increased: (*F*(_10,429)_ = 7.480, *P* < 0.001). Only males receiving a dose of 50 Gy in normoxic conditions and males receiving 100 Gy or 150 Gy in severe hypoxic conditions had significantly shorter longevities than the control group (Fig. 2C).

### Male activity

**Fig. 3:**
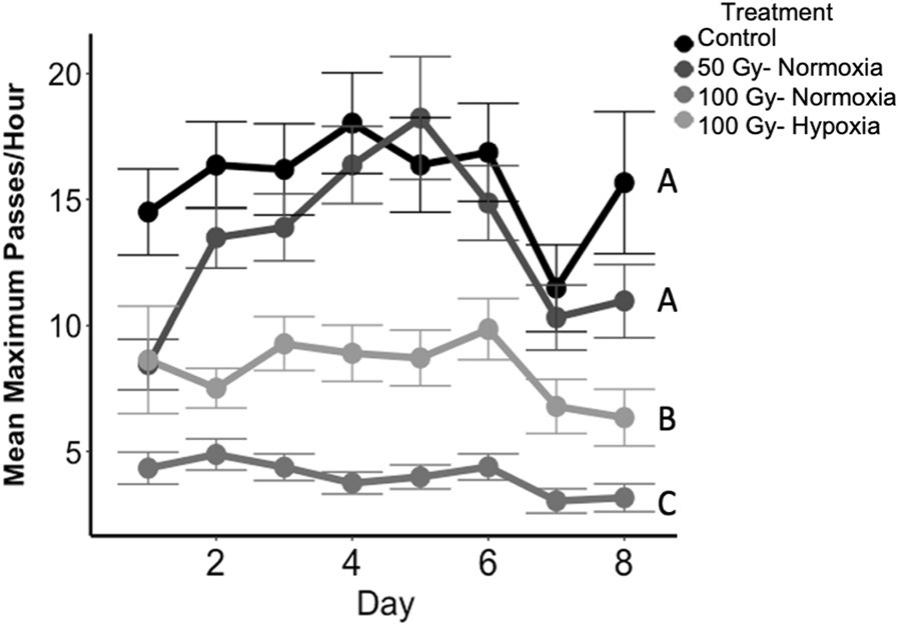
Mean peak daily activity, represented as passes per hour for selected treatment groups. Bars represent standard error; letters indicate significant differences among treatments

Adult male activity did not show any clear trend with age over the duration of monitoring in any dose or atmosphere combination in the first dose range trial. When a linear contrast was performed on the control versus 50 Gy normoxia groups, 100 Gy normoxia group and 100 Gy severe hypoxia group, males dosed to 100 Gy in severe hypoxia were significantly less active than control males but significantly more active than males dosed to 100 Gy in normoxic conditions (F_3,28_ = 53.21, P < 0.001, Fig. 3).

### Effects of male treatment on female fecundity and fertility

**Fig. 4:**
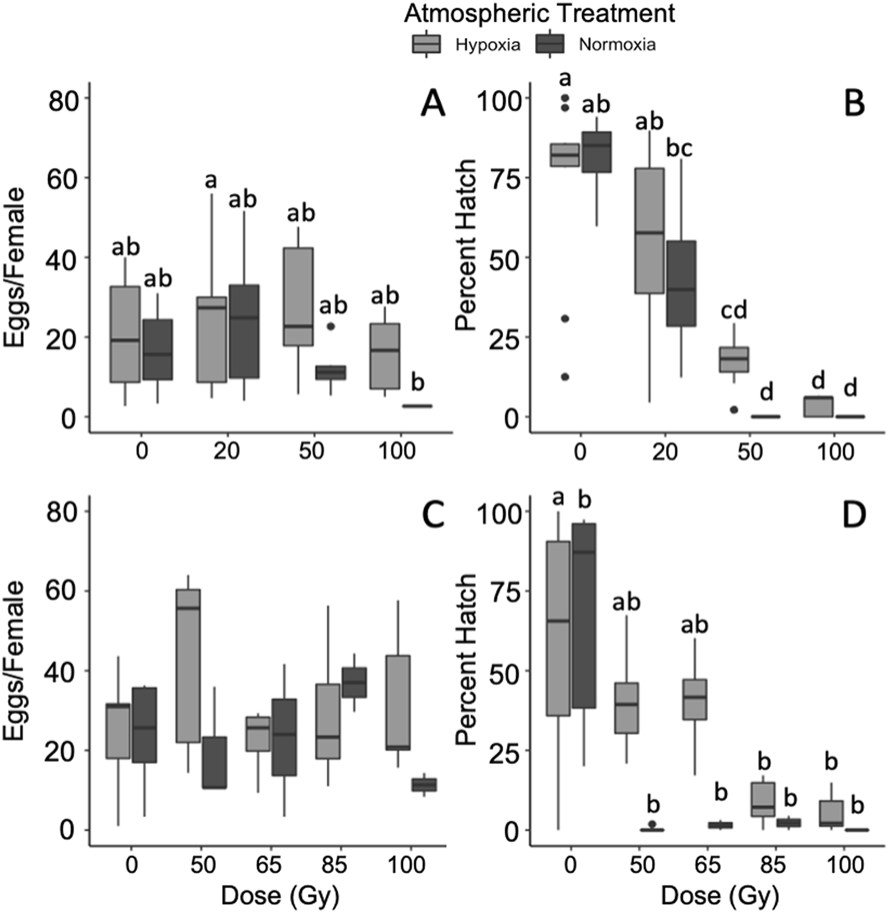
**A** Only females mated with males dosed to 100 Gy under normoxia laid significantly fewer eggs than other groups in the first dose range experiment. **B** There was no clear relationship between radiation dose of males and female egg production when males were dosed under severe hypoxia, while egg production declined in females as male dose increased under normoxic conditions in the second dose range experiment (**C** and **D**)

After Dixon’s Q tests, one replicate of 100 Gy normoxia and two replicates each of 0 Gy normoxia and 100 Gy hypoxia were excluded from analysis in the first dose range. In the first dose range trial (0, 20, 50 and 100 Gy), radiation dose (*F*_1,87_ = 11.33, *P* = 0.001, *η*^2^ = 0.11 [0.03–0.22]) significantly impacted the number of eggs laid by females, while atmospheric conditions (*F*_1,87_ = 3.63, *P *= 0.059, *η*^2^ = 0.04 [0.00–0.12]) of treated males had only a marginally significant impact on the number of eggs laid by females (Fig. 4A). There was no interaction between dose and atmospheric conditions (*F*_1,87_ = 0.84, *P* = 0.361). Male fertility significantly decreased at high doses of radiation in the first dose range trial (*F*_1,51_ = 72.80, *P* < 0.001, *η*^2^ = 0.56 [0.41—0.67]). Atmospheric conditions alone did not significantly impact male fertility (*F*_1,51_ = 1.22, *P* = 0.275, *η*^2^ < 0.001 [0.00–0.10]) but there was a significant interaction between dose and atmospheric conditions (*F*_1,51_ = 4.16, *P* = 0.047, *η*^2^ = 0.03 [0.00–0.15]) wherein at high doses males irradiated in hypoxia had greater fertility than males treated in normoxia (Fig. 4B).

In the second dose range trial, two replicates of 100 Gy in hypoxia and one replicate each of 50 Gy normoxia, 85 Gy hypoxia and 100 Gy normoxia were removed from analysis as outliers. In the second dose range trial (0, 20, 50, 65, 85 and 100 Gy), neither radiation dose (*F*_1,38_ = 0.03, *P* = 0.874) of males nor atmospheric conditions (*F*_1,38_ = 0.06, *P* = 0.884) prior to and during irradiation impacted the number of eggs laid by females (Fig. 4-B). In the second dose range trial, there was also no interaction between dose and atmospheric condition (*F*_1,38_ = 0.02, *P* = 0.886). Only radiation dose (*F*_1,39_ = 44.03, *P* < 0.001, *η*^2^ = 0.51 [0.32–0.64]) strongly affected male fertility in the second dose range trial. Neither atmospheric condition (*F*_1,39_ = 3.05, *P* = 0.088) nor an interaction between dose and atmosphere (*F*_1,41_ = 1.77, *P* = 0.314) had any impact on male fertility (Fig. 4C, D).

**Fig. 5:**
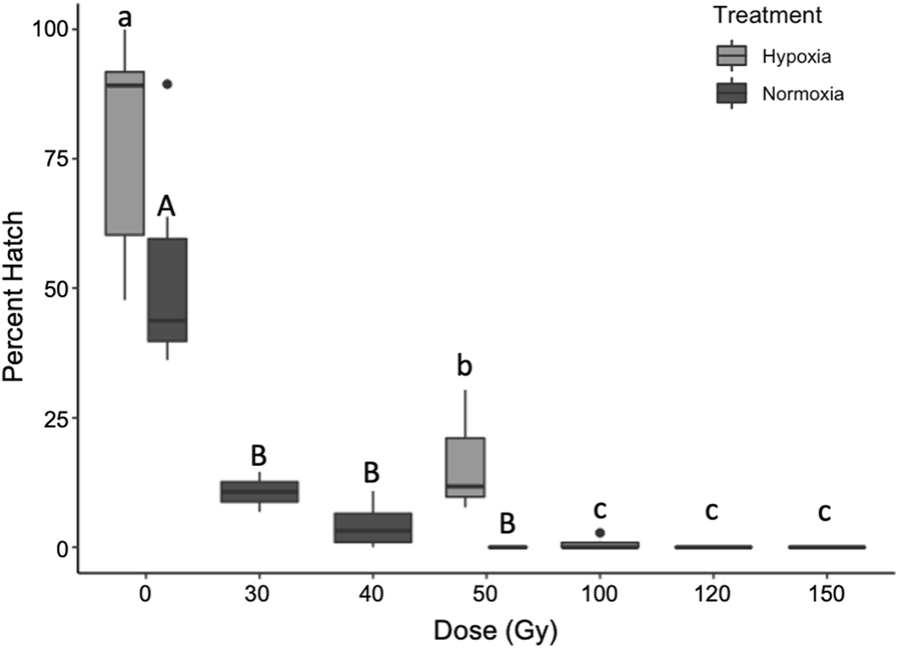
Percent hatch of eggs produced by females mated with males under different atmospheric conditions during irradiation across increasing doses. Letters indicate significant differences among groups within atmospheric treatment. Numbers above bars indicate average percent hatch

**Fig. 6:**
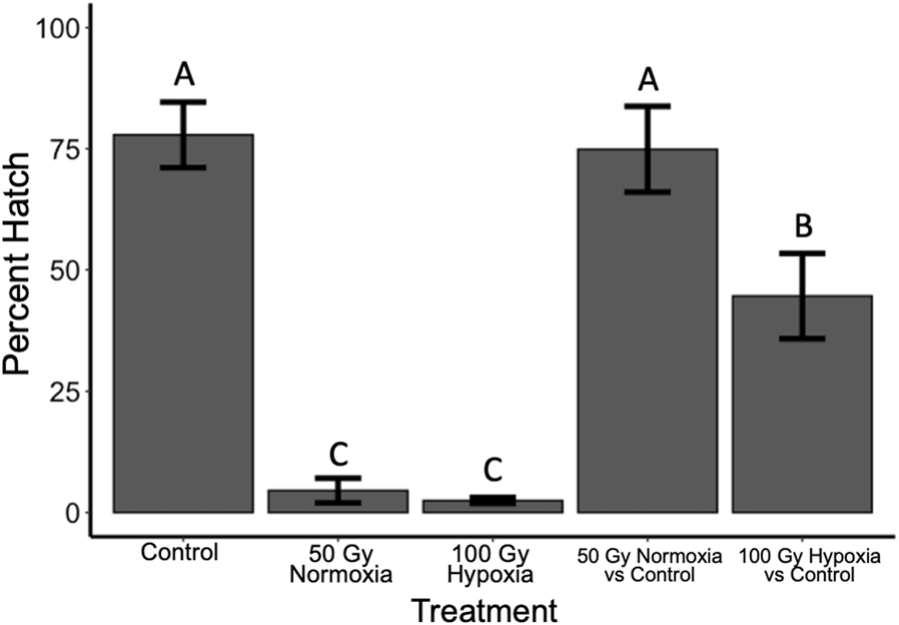
Percent hatch (± SE) of eggs in cages containing 2:1 overall ratio of males to females. Competition cages consisted of 1:1:1 sterile male to wild-type male to females. Competition cages with males dosed to 100 Gy under severe hypoxia had a lower percent hatch than competition cages with males dosed to 50 Gy under normoxia

In the third dose range trial, males irradiated in severe hypoxic conditions retained significantly greater fertility than males irradiated in normoxic conditions at doses of ≤ 50 Gy (Fig. 6). Based on ANOVA models sterility approached 100% at 43.8 Gy (*F*_1,11_ = 33.99, *P* < 0.001, Adj. *R*^2^ = 0.733) and 109.4 Gy (*F*_1,16_ = 56.44, *P* < 0.001, Adj. *R*^2^ = 0.765) for males irradiated in normoxic conditions and under severe hypoxia, respectively (Fig. 5).

### Sterile male competitiveness

Across all treatments, there were no significant differences in the number of eggs produced (*F*_4,24_ = 0.33, *P* = 0.857). The average percent hatch of eggs from females mated with untreated wild-type males was 77.8 ± 6.8%. When caged without wild-type males, cages containing males irradiated under normoxic conditions to a dose of 50 Gy and cages containing males irradiated under severe hypoxia to 100 Gy were not significantly different from each other with hatching percentages of 4.5 ± 2.6% and 2.5 ± 0.6% of eggs hatching, respectively. When caged at a 1:1 ratio with wild-type males, 50 Gy normoxia males were poor competitors because there was no detectable reduction in egg hatch percentage from control cages, 77.8 ± 6.8% and 74.9 ± 8.9% for control and 50 Gy normoxia competition cages, respectively. In 1:1 competition cages between wild-type males and males irradiated under severe hypoxia to 100 Gy, percent hatch of eggs was significantly lower at 44.6 ± 8.8%, compared to control cages (*F*_4,20_ = 25.1, *P* < 0.001) (Fig. 6). When we compare how much sterility was introduced in cages where males treated at 50 Gy in normoxia were competed against unirradiated males vs. treatments in which males irradiated at 100 Gy in anoxia were competed against unirradiated males with a linear contrast, percent hatch was reduced by nearly half in competition cages with males dosed to 100 Gy under anoxic conditions (44.6 ± 8.8%) compared to competition cages with 50 Gy dosed males in normoxia (74.9 ± 8.9%, *t* = 2.40, *P* = 0.026). Fried’s competiveness index (± SE) was calculated as 0.223 ± 0.29 and 1.24 ± 0.54 for males treated with 50 Gy of radiation in normoxic conditions and males treated with 100 Gy in severe hypoxia, respectively, although this difference was not significantly different (*t*_8_ = 1.65, *P* = 0.139).

## Discussion

The goal of this study was to determine the impact of hypoxia treatments during sterilization through ionizing radiation on pupal *Aedes aegypti* across several metrics pertinent to SIT, including survival, activity and mating competiveness. Previous experiments have investigated the radioprotective benefits of hypoxia on *Ae. aegypti*. Hallinan and Rai [56] found that pupae irradiated after 0.5 h in hypoxia required a higher dose to reach sterility, but males irradiated after hypoxia exposure were more competitive against fertile males than sterile males irradiated in normoxia. More recent studies have similarly found that exposure to 0.5 h of hypoxic conditions decreases induced sterility in *Ae. aegypti* [57, 59]. However, none of these previous experiments have investigated the overall impacts of hypoxia on sterile male performance metrics including longevity, activity and mating competitiveness. Our experiments found that exposing 24–36-h-old pupae to severe hypoxia for 1 h prior to and during irradiation affected the dose necessary for > 95% male sterility and induced beneficial plastic responses with respect to competitiveness against non-irradiated males in *Ae. aegypti*.

Radiation treatments performed in severe hypoxia greatly improved dosed male survival. In the first two longevity experiments, the longevity of males exposed to severe hypoxia did not differ from untreated control males at any dose of radiation, while there was a clear decline in longevity for normoxia-treated males as radiation dose increased. This decline in performance was noted at doses as low as 50 Gy in males irradiated in normoxia, which is currently the dose and atmosphere used in a SIT project underway in St. Johns County, FL. Most importantly, in our mating competition experiments, males dosed to 100 Gy in severe hypoxia were significantly more effective in reducing egg hatch when caged with wild-type males than males dosed to 50 Gy in normoxic conditions. The results from this work suggest that hypoxia exposure can improve male performance despite the increase in radiation dose required for adequate sterilization.

Our activity assay was designed to be a predictor of potential field performance, but males were largely inactive during trials. Although we did see a pattern in activity, other experiments conflicted with the results. Despite observed lower movement in our activity trials, 100 Gy-dosed males in hypoxia were much more competitive against wild-type males than 50-Gy dosed males in normoxia in caged competition experiments. It seems more likely that the lack of activity was largely driven by an absence of stimuli during activity assays. It is critically important to develop a reliable metric for flight and dispersal capacity for mosquito SIT. Culbert et al. [60] developed a flight ability device for mosquitoes to serve as a quality control assay for sterile male performance, but it is unclear how well the results from the assay translate to the field. Quality control assays that are predictive of the dispersal ability of released sterile insects are frequently implemented for SIT programs in Lepidopteran and other Dipteran pests [49, 61].

Severe hypoxia durations of 1 h did induce a hormetic response that protected fertility against radiation in males. Much higher doses of radiation were necessary to achieve similar sterility in males irradiated in severe hypoxia compared to males irradiated in normoxic conditions. Apart from activity experiments, doubling the dose of radiation from 50 Gy in normoxia to 100 Gy in severe hypoxia still had noticeable benefits, specifically longer lifespan and increased mating competitiveness, while achieving suitable sterility levels for population reduction. There is ongoing debate about the appropriate sterility level to target while maintaining male competiveness [62, 63]. Achieving 100% sterility through radiation often requires a dose high enough to induce severe deleterious effects on performance metrics such as mate-finding and dispersal [64, 65]. For example, Rull et al. [62] found that achieving only 95% sterility in *Anastrepha obliqua* males resulted in higher performing males than 100% sterile ones and more capable at suppressing populations in the field despite 5% residual fertility. Our competiveness experiments found similar results, with our severe hypoxia-treated males dosed at 100 Gy having around 5% residual fertility, but being significantly more effective at reducing egg hatch percentage when competing with wild-type males than 100% sterile males irradiated in normoxic conditions. A high mating competitiveness has been previously suggested to be more important for mosquito SIT programs than 100% sterility [31, 33].

Incorporating severe hypoxia treatments into existing production plans for mass rearing and sterilization of mosquitoes requires a few considerations. First, a suitable container for packing and flushing with nitrogen must be determined, factoring in the number and density of mosquitoes that need to be irradiated as well as meeting physical constraints of the irradiator [66]. Mosquitoes should be kept in an area within the irradiator that receives a uniform dose [32]. The material of the container and the source of ionizing radiation (gamma vs. high powered x-ray) could also impact the response to hypoxia exposure during radiation treatments [31, 32, 66]. If multiple runs of irradiators are needed to produce a sufficient supply of sterile males, it will be important to stagger the purging and reperfusion of oxygen in containers to ensure uniform durations of hypoxic conditions on pupae during radiation treatments.

The method of creating a hypoxic environment is also important to consider. In the present experiments we placed pupae on moist filter paper and flushed air-tight containers with nitrogen to remove as much water as possible. Other studies have kept pupae in water and created a hypoxic environment by forcing out dissolved O_2_ by sparging it with nitrogen [57, 59]. Water is known to attenuate gamma and x-rays, so it would be necessary to alter the adjust irradiation timing to ensure the pupae achieve the intended absorbed dose [31, 32, 59].

If hypoxia treatment prior to and during irradiation is to be considered for mosquito SIT, future studies focusing on the performance of hypoxia-treated sterile males in field conditions will be needed. Although our hypoxia-treated sterile males lived longer and were more competitive than males sterilized in normoxia in laboratory conditions, additional environmental stressors could either negate or enhance differences between atmospheric treatments. Laboratory-reared insects are typically less resilient in field conditions compared to wild conspecifics [34, 43, 67]. Exposure to reduced oxygen environments has been found to improve field performance in the Mediterranean fruit fly, *Anastrepha suspensa*, and in the moth, *Cactoblastis cactorum* [44, 68], but this has yet to be tested in mosquitoes. With the observation of lower activity in hypoxia-treated males dosed to 100 Gy than males dosed to 50 Gy in normoxia, mark-release-recapture studies should be conducted to determine whether lower activity in laboratory conditions translates into reduced dispersal capacity. Field longevity and dispersal capacity are critical factors in the success of a SIT program [69–71]. If mark-release-recapture studies suggest better field dispersal and longevity for hypoxia-irradiated male mosquitoes, then pilot tests for suppression that measure field mating competitiveness could follow [33].

Another focus for future work could be to determine the impacts of hypoxia treatments on adult mosquitoes. Research on multiple species has found that some insects suffer reduced somatic tissue damage when sterilized in the adult stage [66, 72, 73]. This has been found true in some mosquito studies [66, 74], but research on sterilizing adult mosquitoes in reduced oxygen atmospheres is scant. Hallinan and Rai [56] found that both pupae and adult male *Aedes aegypti* subjected to 0.5 h of hypoxia significantly improved mating competitiveness compared to conspecifics irradiated in normoxia. Contrastingly, El-Gazzar et al. (1983) found that adult *Culex quinquefasciatus* did not receive any significant irradiation protection from hypoxic conditions during irradiation and that the radioprotective effects of hypoxia on pupae or adults did not persist once reaching high sterility levels.

Irradiating males as young adults instead of pupae may confer several benefits for a SIT program. Irradiating adults can reduce somatic tissue damage that could impair performance of sterile males as well as production benefits such as a facility staff having a wider time window for irradiating adults while achieving the same sterility levels compared to pupae, which are much more age sensitive to radiation exposure [75–77]. From a practical standpoint, however, adults are much more fragile and mobile than pupae, so handling adults through the sterilization process may be difficult. It may be more practical to irradiate pupae in hypoxia to improve quality rather than handling adult mosquitoes.

## Supplementary Information

**Additional file 1: Figure S1.** Mean peak daily activity, represented as passes per hour in the most active hour, for all treatment groups. The dots represent the means, bars represent the standard errors, and asterisks indicate a significant difference between the atmospheric treatment groups within a radiation dose. A significant effect of atmospheric treatment on activity in this assay was only detected in our 100 Gy radiation-dose group.

## Data Availability

The datasets used and/or analyzed during the current study are available from the corresponding author on reasonable request.
