# Supplementary figures and images for: Does severe hypoxia during irradiation of Aedes aegypti pupae improve sterile male performance?
Source: Parasit Vectors. 2022 Nov 28;15:446. doi: 10.1186/s13071-022-05577-0 (PMC9706925; doi:10.1186/s13071-022-05577-0)

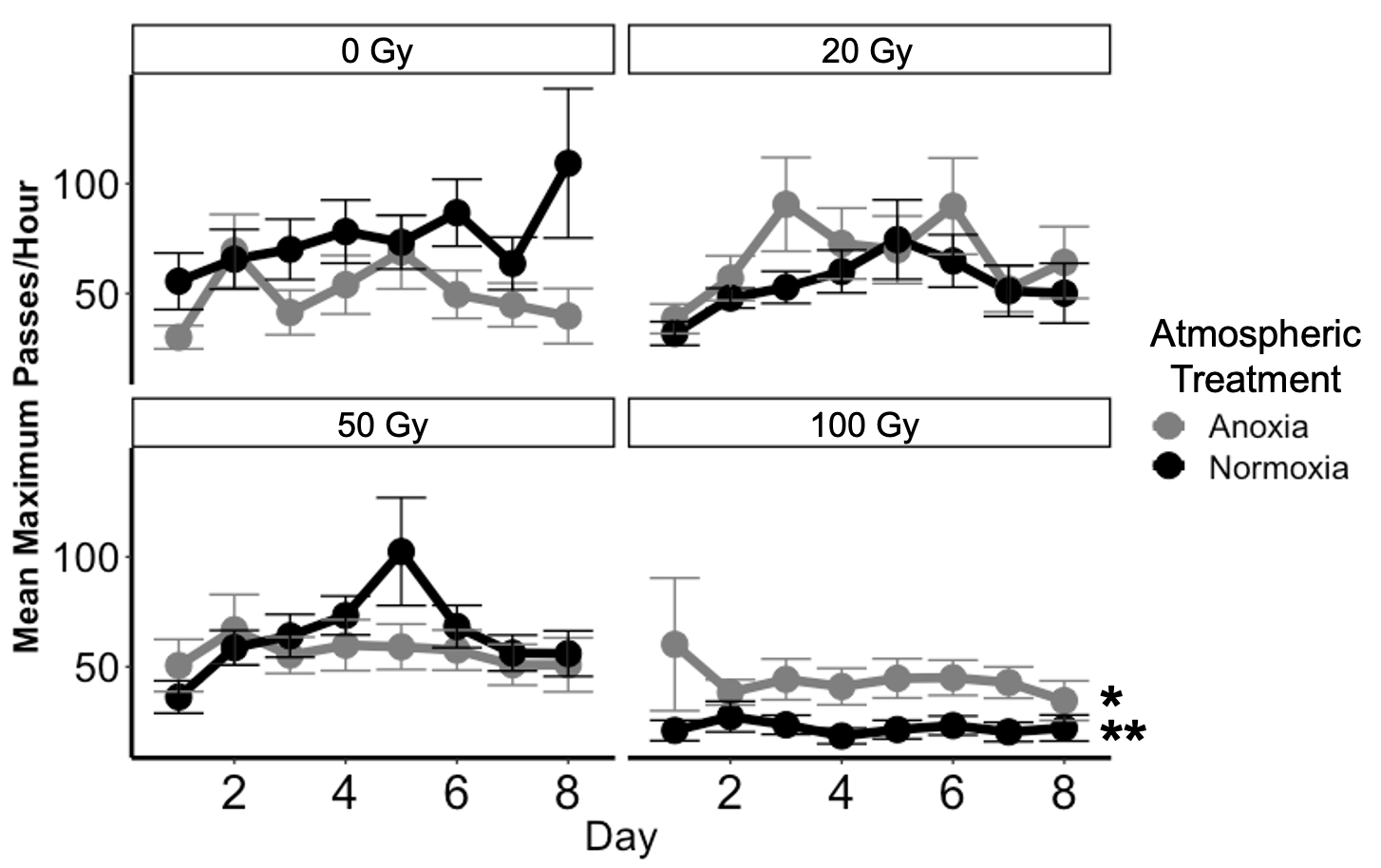

Supplement: Supplementary file 1 — Additional file 1: Figure S1. Mean peak daily activity, represented as passes per hour in the most active hour, for all treatment groups. The dots represent the means, bars represent the standard errors, and asterisks indicate a significant difference between the atmospheric treatment groups within a radiation dose. A significant effect of atmospheric treatment on activity in this assay was only detected in our 100 Gy radiation-dose group. [file 13071_2022_5577_MOESM1_ESM.tiff]
